# Induced Extracellular Ice Nucleation Protects Cocultured Spheroid Interior and Exterior during Cryopreservation

**DOI:** 10.1021/acsbiomaterials.4c00958

**Published:** 2024-09-24

**Authors:** Yanan Gao, Akalabya Bissoyi, Qiongyu Guo, Matthew I. Gibson

**Affiliations:** † Department of Chemistry, University of Warwick, Coventry CV4 7AL, United Kingdom; ‡ Division of Biomedical Sciences, Warwick Medical School, University of Warwick, Coventry CV4 7AL, United Kingdom; § Department of Chemistry, University of Manchester, Oxford Road, Manchester M13 9PL, United Kingdom; ∥ Manchester Institute of Biotechnology, University of Manchester, 131 Princess Street, Manchester M1 7DN, United Kingdom; ⊥ Department of Biomedical Engineering, Southern University of Science and Technology, Shenzhen, Guangdong 518055, China

**Keywords:** 3D spheroid, coculture, cryopreservation, ice nucleation, cell-based models

## Abstract

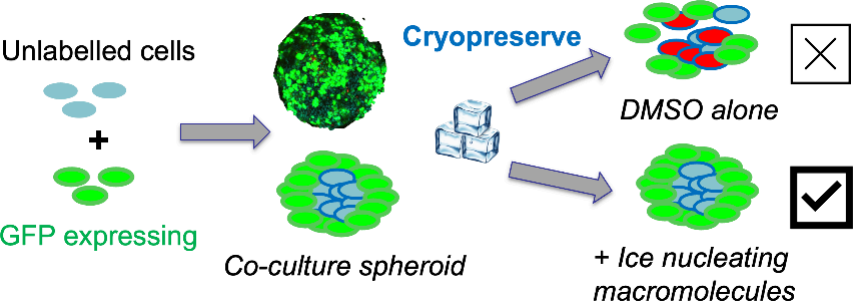

Spheroids and other 3D cellular models more accurately recapitulate physiological responses when compared to 2D models and represent potential alternatives to animal testing. The cryopreservation of spheroids remains challenging, limiting their wider use. Standard DMSO-only cryopreservation results in supercooling to low subzero temperatures, reducing viability, shedding surface cells, and perforating spheroid interiors. Here, cocultured spheroids with differentially labeled outer cell layers allow spatial evaluation of the protective effect of macromolecular ice nucleators by microscopy and histology. Extracellular nucleation is shown to reduce damage to both interior and exterior regions of the spheroids, which will support the development of “off-the-shelf” 3D models.

## Introduction

Two-dimensional cell cultures (monolayers) are widely used in drug screening processes and are suitable for high-throughput and automated methods in toxicology.^1^ However, 2D models cannot replicate *in vivo* niches due to the lack of extracellular matrix and limited intercellular communication. Spheroids, three-dimensional aggregates of cells, can recapitulate several aspects of *in vivo* tissue architecture and can be assembled from a range of cell types.^2−4^ Their relevance spans from basic biological studies to advanced applications in drug discovery, tissue engineering, and regenerative medicine. These *in vitro* models also contribute to the target of the 3R’s (reduction, refinement, and replacement) for animal testing due to their superior predictive power when compared to 2D monolayers^5^ and the acknowledged challenges of predicting human physiological responses from nonhuman models.^6^ For example, HepG2 (immortalized hepatocyte) spheroids accurately predicted *in vivo* (rat) lethal plasma levels, matching the performance of a 2D primary cell model.^7^ Despite these benefits, spheroids are less widely used than 2D models due to the additional time, resources, and skill sets required in assembly. A practical solution is to develop cryopreservation strategies which facilitate “off the shelf” use of spheroids. These would require minimal preparation for a user, just a simple thawing process. This would also aid reproducibility.^8^

Dimethyl sulfoxide (DMSO) is the most widely used cryoprotective agent (CPA) for mammalian cells and is routinely used especially for suspension (e.g., in cryovials) storage of cell lines.^9−11^ The preservation of primary cells or complex 3D models, including spheroids and tissues, present unique hurdles due to their complex architecture, cellular heterogeneity, and their physical size which present various permeation barriers. The challenges of nutrient and cryoprotectant transport as well as the propagation of ice crystals and intercellular contacts need to be overcome. The reformulation of CPA composition,^12^ or targeted additives such as ROCK (rho-associated kinase) inhibitors or osmolytes can increase post-thaw recovery.^13−16^ As an alternative to small-molecule CPAs, macromolecular cryoprotectants that address additional biophysical modes of cryoinduced damage have emerged. For example, polyampholytes^17^ based on lysine^18^ or synthetic polymer^19^ backbones have found widespread use in cell storage, potentially aiding cellular dehydration and ion transport.^20^ Polyampholytes can improve the post-thaw recovery of spheroids^21^ but do not address the issue of supercooling (delayed ice nucleation). During the cooling stage of cryopreservation, the CPA solution can supercool, leading to freezing (nucleation) occurring as low as −20 °C.^22,23^ This delay leads to cells being exposed to cytotoxic CPAs for longer and reduces cell dehydration (compared to a warm temperature nucleation), which leads to an overall reduction in recovery. Induced nucleation can overcome this challenge by increasing the freezing temperature closer to zero and has been demonstrated to increase spheroid recovery^15,24,25^ and reduce cell–cell contact damage.^26^ Despite these advances, it is not clear how chemically induced ice nucleation in the extracellular media (compared to physical stimulation) leads to protection of the entire spheroid and retention of all cell–cell contacts and if surface-exposed cells are protected more or less than in the interior. Percentage cell recovery, while useful, does not capture the heterogeneity within/between spheroids. Understanding the impact of nucleation over the entire spheroid is crucial to develop cryopreservation tools for increasingly complex multicellular organoids where spatial distribution of cells is crucial.

Here we use a coculture spheroid model where the outermost cells stably express a green fluorescent protein (GFP) to enable spatial resolution of post-thaw spheroid integrity. Ice nucleation is triggered using water-soluble nucleators isolated from pollen^23,27,28^ and is shown to increase the recovery of spheroids. Using a combination of morphological analysis, fluorescence microscopy, and histology, we show that chemically induced ice nucleation protects the entirety of the spheroid structure. In the absence of nucleation, cells at the periphery of the spheroid are shed, and the interior shows significant damage and perforation. This demonstrates how chemically induced nucleation in extracellular media has a protective impact over the entire 3D structure.

## Results

Our experimental design used cocultures of spheroids where the outer layer was A549 (human Caucasian lung carcinoma) cells stably expressing GFP (green fluorescent protein), with a core of either A549 or HepG2 (human hepatocellular carcinoma) cells (Scheme S1A). By labeling the outer cells, the post-thaw recovery of the spheroids, with particular reference to surface cell-shedding, can be critically evaluated when chemically induced extracellular ice nucleation (IN+) is used (Scheme S1B). This is crucial as supercooling (delayed nucleation) is known to cause conventional 2D cell monolayers (on tissue culture plastic) to detach, leaving an incomplete layer. By labeling the periphery of spheroids, we can identify this effect in 3D models, additionally providing more detail on how materials which induce nucleation can benefit complex cell model cryopreservation.

**Figure 1:**
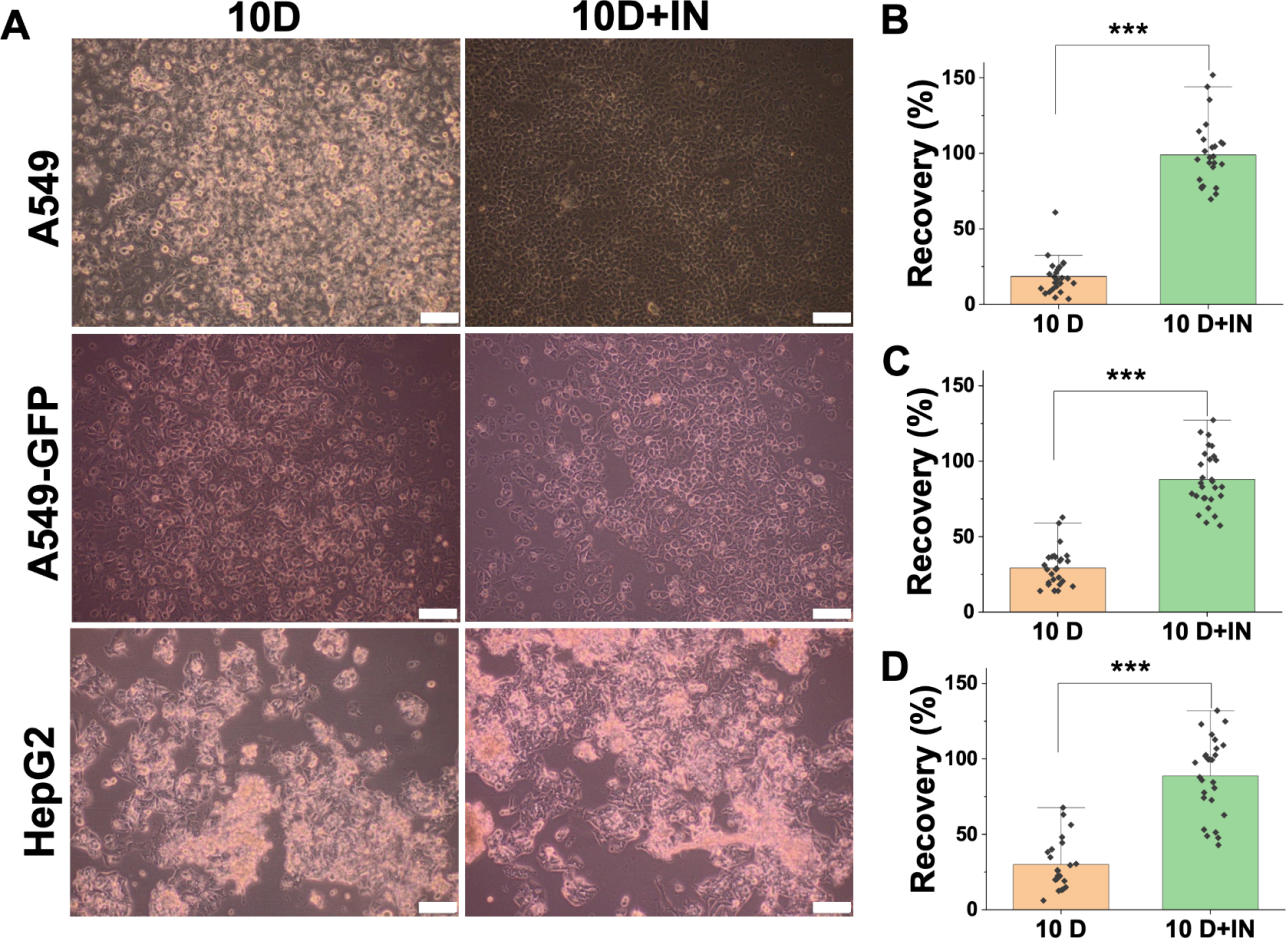
Post-thaw recovery of 2D cell monolayers using DMSO alone (10D) or with induced ice nucleation (10D+IN). (A) Optical microscopy of cells 24 h post-thaw. (B) Post-thaw cell recovery of A549 cells. (C) Post-thaw cell recovery of A549-GFP cells. (D) Post-thaw cell recovery of HepG2 cells. Scale bar: 100 μm. Number of technical and biological replicates ≥3. ***P < 0.001.

As a first test, each cell line in isolation was cryopreserved in 10% DMSO as a monolayer to ensure that the induced ice nucleation (IN+) provided an increase in post-thaw recovery, particularly for the engineered A549-GFP which had not been cryopreserved in this manner before (microscopy in Figure S1). The 24 h (to avoid false positives^29^) post-thaw cell recovery with and without nucleation is shown in Figure 1. In all cases, the induced nucleation led to significant increases in post-thaw recovery from 20% to almost 100%, in line with previous reports.^25,30,31^

**Figure 2:**
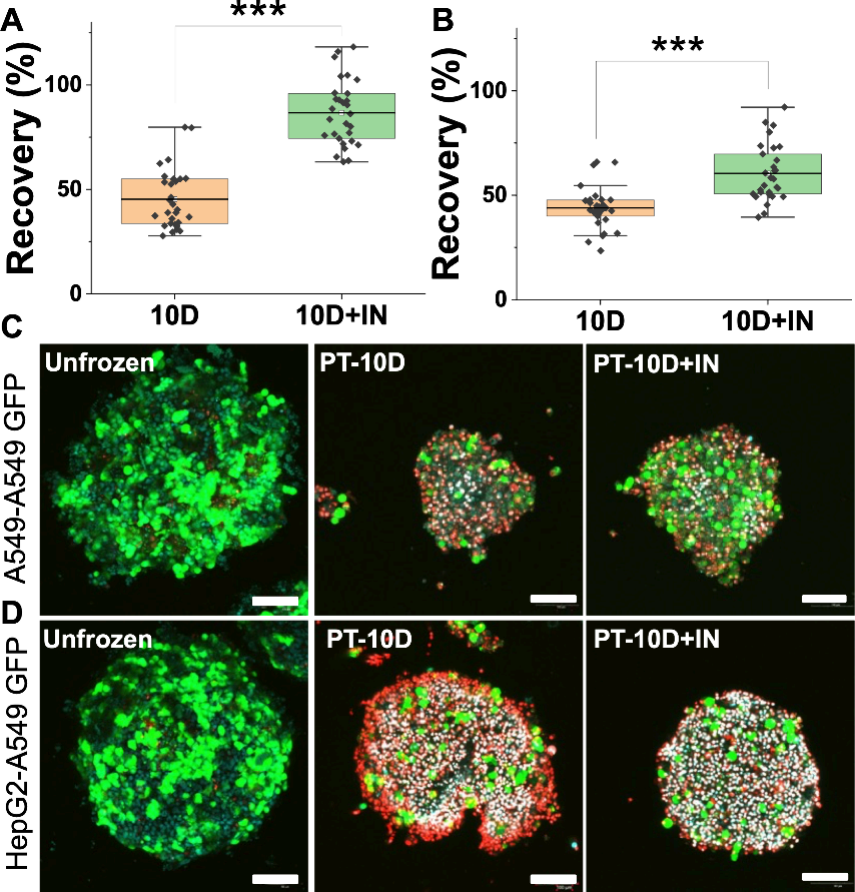
Post-thaw (24 h) recovery of cocultured spheroids. (A) Recovery of A549–A549-GFP spheroids. (B) Recovery of HepG2–A549-GFP spheroids. (C) Confocal microscopy of A549–A549-GFP. (D) Confocal microscopy of HepG2–A549-GFP before and after cryopreservation with indicated conditions. PT = post thaw; 10D = 10% DMSO; +IN = induced ice nucleation. Green = A549-GFP; Red = dead (ethidium homodimer-1, EI); white = (overexposed) Hoechst 33342. Scale bar: 100 μm. Number of technical and biological replicates ≥3. ***P < 0.001.

Following the confirmation that extracellular ice nucleation could protect the relevant cell lines, spheroids were produced using agarose molds seeded with 1000–8000 cells (Figure S2A). Recovery was measured by a metabolic viability assay (WST-1) of the whole spheroid compared to unfrozen controls. Initial screening showed that post-thaw recovery was highest for smaller spheroids with diameters around 200 μm, compared to larger spheroids of 400 μm; this was as expected and in line with our previous reports^15,21,25^ (Figure S2 B-D). Spheroids with an outer layer of A549-GFP cells and inner core of either A549 or HepG2 cells were prepared (Figure S3, ∼400 μm) and cryopreserved in either 10% DMSO (standard condition) or 10% DMSO plus induced ice nucleation, IN+. The spheroids were thawed and then evaluated for post-thaw recovery after 24 h using the WST-1 (metabolic activity) assay. Figure 2A,B shows the impact of induced ice nucleation which in both cases produced increases in the post-thaw cell recovery. The A549–A549-GFP spheroids recovered better (>80%) compared to the HepG2–A549-GFP spheroids (60%). We attribute this to previous observations that HepG2 cells tend to recover less well post-thaw than A549 cells^25^ (which are a very robust model) and also the two different cell types in this model. To reiterate, our aim is not to optimize recovery (such as adapting the freezing rate and other associated tools) but to probe how nucleation protects 3D cell models. With the supplement of IN into 10% DMSO solution, the nucleation temperature increased to −9.25 °C compared to −15.77 °C (10% DMSO alone) (Figure S4). To visualize the protective mechanism, confocal microscopy was also used. In Figure 2C,D, green represents the A549-GFP cells, and red are dead cells (ethidium homodimer-1, EI). Nuclei were labeled by Hoechst 33342, which due to the exposure results in a white coloration which was not corrected in the image to minimize processing. In these initial experiments, for unfrozen controls, the green A549-GFP outer layer is clearly visible. After cryopreservation in 10% DMSO, both types of spheroids exhibited distorted shapes with a loss in the green (A549) cells and extensive dead/membrane compromised cells (shown in red). In contrast, when cryopreservation was performed with induced ice nucleation, there was a significant reduction in “red” labeled cells. With induced ice nucleation, there was less, but not zero, shedding of the outer A549 cells (Figures S5 and S6). This confirms that the cell detachment from the spheroid surface is a key mode of damage. This agrees with observations on 2D monolayer freezing that cell detachment,^30^ not just intrinsic cell damage, is a major stressor during cryopreservation.

**Figure 3:**
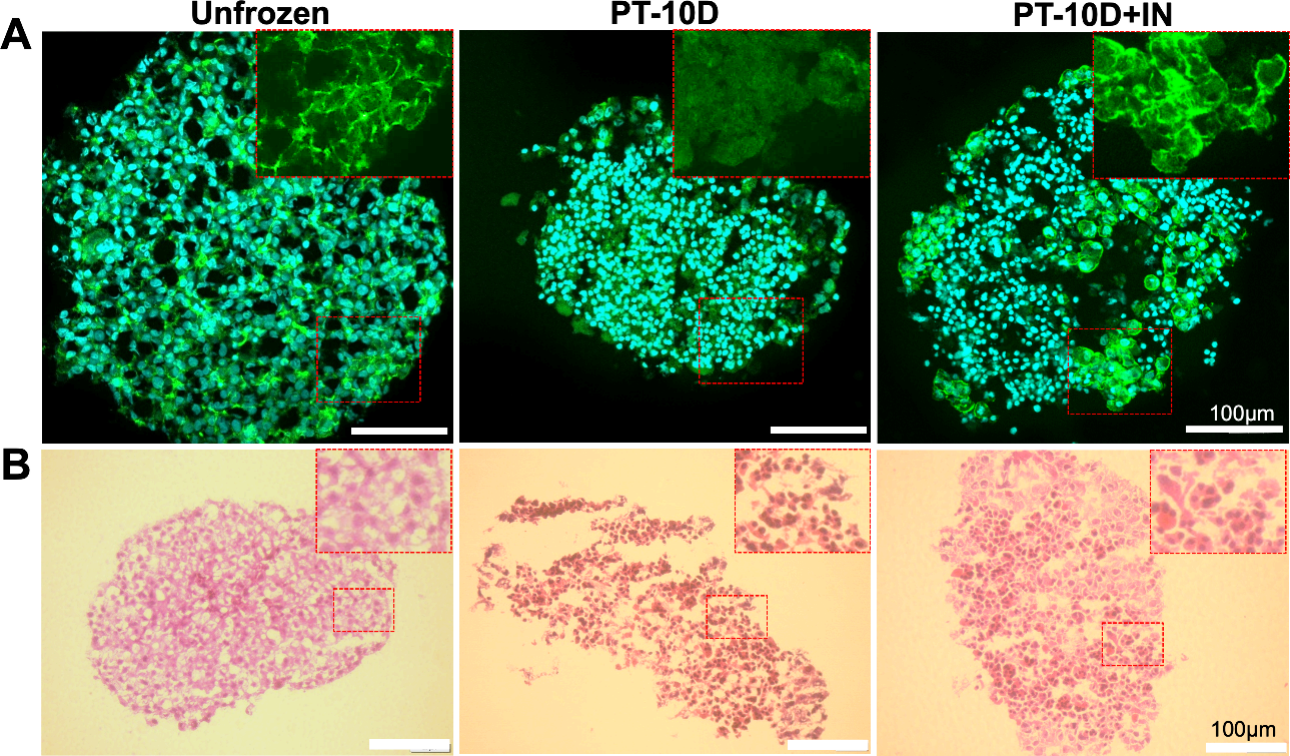
Post-thaw (24 h) recovery of HepG2–A549-GFP cocultured spheroids. (A) Confocal microscopy of HepG2–A549-GFP before and after cryopreservation. Green = F-actin (Incitrogen ActinGreen 488), Blue = Nuclei (Hoechst 33342). (B) H&E staining images of HepG2–A549-GFP spheroids. PT = post thaw; 10D = 10% DMSO; +IN = induced ice nucleation. Scale bar: 100 μm. Red dash line box: zoomed in area.

Further characterization was undertaken by higher resolution imaging taking 10 μm slices through the spheroid, Figure 3. Based on F-actin staining, there was clearly less actin polymerization (post-thaw) when 10% DMSO alone was used, compared to when induced ice nucleation was employed (judged by intensity of fluorescence) (Figure S7 and S8). Actin depolymerization may correlate with intracellular ice nucleation^21^ and is known to cause damage post-thaw.^32^ Therefore, these staining results confirm the hypothesis that inducing ice nucleation at warmer temperatures aids cellular dehydration (compared to supercooling which occurs in standard DMSO-only cryopreservation) and, thus, reduces total intracellular ice.^25,30,33^ Histology was also undertaken on the spheroids with hematoxylin and eosin (H&E) staining, Figure 3B (and addition images in Figure S9). Fresh spheroids and those cryopreserved with ice nucleation both showed retained morphology, complete cellular contacts, and evenly distributed cells without large “gaps”, which would have indicated detachment had occurred. In contrast, spheroids which were cryopreserved in 10% DMSO alone exhibited less regular arrangements and lacked strong internal cohesion in their cell-to-cell contacts. Additionally, there was a noticeable presence of interstitial spaces between the individual cells. Most cells were apoptotic cells with a nuclear condensation, which showed darker results (stained strongly with hematoxylin).

We have previously shown that for tight-junction forming cell monolayers (Caco-2) cells, induced ice nucleation leads to differential protein expression (using whole cell proteomics), post-thaw.^26^ This was attributed to a retention of specific cellular pathways in this cell type, which are not present in A549 cells. Volcano plots and pathway mappings (Figures S10–12) revealed no significant differences when induced nucleation was used despite the increased cell recovery. This would imply that the impact is purely biophysical and not biochemical in this particular case, but a deeper proteomic/pathway analysis is required to confirm this. These data clearly demonstrate how inducing nucleation in the extracellular environment leads to protection throughout an entire spheroid and not just of the surface-exposed cells. These observations validate the use and need to discover new ice nucleating macromolecular additives to aid the storage, banking, and transport of complex cell models.

## Conclusions

Here we have demonstrated that chemically induced extracellular ice nucleation leads to an increase in post-thaw recovery of cryopreserved multilayer spheroids by reducing the total amount of surface-exposed cell shedding as well as protecting the interior. Chemically programmed ice nucleation was achieved by using soluble ice nucleating macromolecules extracted from pollen. Interior and exterior localization of cells within spheroids was achieved using cocultures where the outermost (solvent exposed) layers were made using cells stably expressing green fluorescent protein allowing separation of recovery between interior and exterior cells. Induced nucleation increased overall cell recovery (judged by metabolic assays) but also led to the retention of spheroid culture post-thaw.

Using high resolution imaging, it was seen that induced nucleation also resulted in cells with increased actin polymerization, which has been linked to reduced intracellular ice formation during cryopreservation. However, a causal link has not yet been fully established. Furthermore, histology clearly showed that without induced nucleation, the supercooling leads to damage of the spheroid overall structure with significant fragmentation, which was not seen when ice was nucleated. Overall, this demonstrates that an ice nucleator that is external to the spheroid is able to protect the entire spheroid, not just the solvent-exposed exterior, by preventing supercooling. This also demonstrates how labeled cocultures can be used to selectively probe the cryobiological mechanism of damage beyond simply measuring net recovery. These results will help develop banking strategies for spheroids to enable their use in drug development and related fields. It also helps guide the discovery of further cryoprotectants targeting specifically modes of physical damage that occur during cryopreservation.

## Supporting Information Available

Detailed experimental procedures as well as additional data including confocal microscopy images and proteomics data (PDF)

## References

[ref1] Sun D.; Gao W.; Hu H.; Zhou S. Why 90% of Clinical Drug Development Fails and How to Improve It?. Acta Pharm. Sin. B 2022, 12 (7), 3049–3062. 10.1016/j.apsb.2022.02.002. 35865092 PMC9293739

[ref2] Griffith L. G.; Swartz M. A. Capturing Complex 3D Tissue Physiology in Vitro. Nat. Rev. Mol. Cell Biol. 2006, 7 (3), 211–224. 10.1038/nrm1858. 16496023

[ref3] Turco M. Y.; Gardner L.; Hughes J.; Cindrova-Davies T.; Gomez M. J.; Farrell L.; Hollinshead M.; Marsh S. G. E.; Brosens J. J.; Critchley H. O.; Simons B. D.; Hemberger M.; Koo B.-K.; Moffett A.; Burton G. J. Long-Term, Hormone-Responsive Organoid Cultures of Human Endometrium in a Chemically Defined Medium. Nat. Cell Biol. 2017, 19 (5), 568–577. 10.1038/ncb3516. 28394884 PMC5410172

[ref4] Vinci M.; Gowan S.; Boxall F.; Patterson L.; Zimmermann M.; Court W.; Lomas C.; Mendiola M.; Hardisson D.; Eccles S. A. Advances in Establishment and Analysis of Three-Dimensional Tumor Spheroid-Based Functional Assays for Target Validation and Drug Evaluation. BMC Biol. 2012, 10 (1), 29 10.1186/1741-7007-10-29. 22439642 PMC3349530

[ref5] Stresser D. M.; Kopec A. K.; Hewitt P.; Hardwick R. N.; Van Vleet T. R.; Mahalingaiah P. K. S.; O’Connell D.; Jenkins G. J.; David R.; Graham J.; Lee D.; Ekert J.; Fullerton A.; Villenave R.; Bajaj P.; Gosset J. R.; Ralston S. L.; Guha M.; Amador-Arjona A.; Khan K.; Agarwal S.; Hasselgren C.; Wang X.; Adams K.; Kaushik G.; Raczynski A.; Homan K. A. Towards in Vitro Models for Reducing or Replacing the Use of Animals in Drug Testing. Nat. Biomed. Eng. 2024, 8, 930 10.1038/s41551-023-01154-7. 38151640

[ref6] Olson H.; Betton G.; Robinson D.; Thomas K.; Monro A.; Kolaja G.; Lilly P.; Sanders J.; Sipes G.; Bracken W.; Dorato M.; Van Deun K.; Smith P.; Berger B.; Heller A. Concordance of the Toxicity of Pharmaceuticals in Humans and in Animals. Regul. Toxicol. Pharmacol. 2000, 32 (1), 56–67. 10.1006/rtph.2000.1399. 11029269

[ref7] Fey S. J.; Wrzesinski K. Determination of Drug Toxicity Using 3D Spheroids Constructed from an Immortal Human Hepatocyte Cell Line. Toxicol. Sci. 2012, 127 (2), 403–411. 10.1093/toxsci/kfs122. 22454432 PMC3355318

[ref8] Zanoni M.; Piccinini F.; Arienti C.; Zamagni A.; Santi S.; Polico R.; Bevilacqua A.; Tesei A. 3D Tumor Spheroid Models for in Vitro Therapeutic Screening: A Systematic Approach to Enhance the Biological Relevance of Data Obtained. Sci. Rep. 2016, 6 (1), 19103 10.1038/srep19103. 26752500 PMC4707510

[ref9] Murray K. A.; Gibson M. I. Chemical Approaches to Cryopreservation. Nat. Rev. Chem. 2022, 6, 579–593. 10.1038/s41570-022-00407-4. PMC9294745 35875681

[ref10] Brockbank K.; Taylor M. Tissue Preservation. Advances in biopreservation 2006, 5, 157–196. 10.1201/9781420004229.ch8.

[ref11] Pegg D. E. Principles of Cryporeservation. Methods Mol. Biol. 2007, 368, 39–57. 10.1007/978-1-59745-362-2_3. 18080461

[ref12] Lee J. H.; Jung D. H.; Lee D. H.; Park J. K.; Lee S. K. Effect of Spheroid Aggregation on Susceptibility of Primary Pig Hepatocytes to Cryopreservation. Transplant. Proc. 2012, 44 (4), 1015–1017. 10.1016/j.transproceed.2012.03.009. 22564613

[ref13] Xue W.; Li H.; Xu J.; Yu X.; Liu L.; Liu H.; Zhao R.; Shao Z. Effective Cryopreservation of Human Brain Tissue and Neural Organoids. Cell Reports Methods 2024, 4 (5), 100777 10.1016/j.crmeth.2024.100777. 38744289 PMC11133841

[ref14] Gonzalez-Martinez N.; Gibson M. I. Post-Thaw Application of ROCK-Inhibitors Increases Cryopreserved T-Cell Yield. RSC Med. Chem. 2023, 14 (10), 2058–2067. 10.1039/D3MD00378G. 37859712 PMC10583820

[ref15] Gao Y.; Bissoyi A.; Kinney N. L. H.; Whale T. F.; Guo Q.; Gibson M. I. Proline-Conditioning and Chemically-Programmed Ice Nucleation Protects Spheroids during Cryopreservation. Chem. Commun. 2023, 59, 9086–9089. 10.1039/D3CC02252H. PMC10358716 37401839

[ref16] Bailey T. L.; Wang M.; Solocinski J.; Nathan B. P.; Chakraborty N.; Menze M. A. Protective Effects of Osmolytes in Cryopreserving Adherent Neuroblastoma (Neuro-2a) Cells. Cryobiology 2015, 71 (3), 472–480. 10.1016/j.cryobiol.2015.08.015. 26408850

[ref17] Stubbs C.; Bailey T. L.; Murray K.; Gibson M. I. Polyampholytes as Emerging Macromolecular Cryoprotectants. Biomacromolecules 2020, 21 (7), 7–17. 10.1021/acs.biomac.9b01053. 31418266 PMC6960013

[ref18] Matsumura K.; Hyon S. H. Polyampholytes as Low Toxic Efficient Cryoprotective Agents with Antifreeze Protein Properties. Biomaterials 2009, 30 (27), 4842–4849. 10.1016/j.biomaterials.2009.05.025. 19515417

[ref19] Bailey T. L.; Stubbs C.; Murray K.; Tomas R. M. F.; Otten L.; Gibson M. I. A Synthetically Scalable Poly(Ampholyte) Which Dramatically Enhances Cellular Cryopreservation. Biomacromolecules 2019, 20, 3104–3114. 10.1021/acs.biomac.9b00681. 31268698 PMC6692820

[ref20] Matsumura K.; Hayashi F.; Nagashima T.; Rajan R.; Hyon S.-H. Molecular Mechanisms of Cell Cryopreservation with Polyampholytes Studied by Solid-State NMR. Commun. Mater. 2021, 2 (1), 15 10.1038/s43246-021-00118-1.

[ref21] Bissoyi A.; Tomás R. M. F.; Gao Y.; Guo Q.; Gibson M. I. Cryopreservation of Liver-Cell Spheroids with Macromolecular Cryoprotectants. ACS Appl. Mater. Interfaces 2023, 15 (2), 2630–2638. 10.1021/acsami.2c18288. 36621888 PMC9869333

[ref22] Daily M. I.; Whale T. F.; Partanen R.; Harrison A. D.; Kilbride P.; Lamb S.; Morris G. J.; Picton H. M.; Murray B. J. Cryopreservation of Primary Cultures of Mammalian Somatic Cells in 96-Well Plates Benefits from Control of Ice Nucleation. Cryobiology 2020, 93, 62–69. 10.1016/j.cryobiol.2020.02.008. 32092295 PMC7191264

[ref23] Murray K. A.; Kinney N. L. H.; Griffiths C. A.; Hasan M.; Gibson M. I.; Whale T. F. Pollen Derived Macromolecules Serve as a New Class of Ice-Nucleating Cryoprotectants. Sci. Rep. 2022, 12 (1), 12295 10.1038/s41598-022-15545-4. 35854036 PMC9296471

[ref24] Massie I.; Selden C.; Hodgson H.; Fuller B. Cryopreservation of Encapsulated Liver Spheroids for a Bioartificial Liver: Reducing Latent Cryoinjury Using an Ice Nucleating Agent. Tissue Eng. - Part C Methods 2011, 17 (7), 765–774. 10.1089/ten.tec.2010.0394. 21410301

[ref25] Murray K. A.; Gao Y.; Griffiths C. A.; Kinney N. L. H.; Guo Q.; Gibson M. I.; Whale T. F. Chemically Induced Extracellular Ice Nucleation Reduces Intracellular Ice Formation Enabling 2D and 3D Cellular Cryopreservation. JACS Au 2023, 3 (5), 1314–1320. 10.1021/jacsau.3c00056. 37234117 PMC10207112

[ref26] Bissoyi A.; Gao Y.; Tomás R. M. F.; Kinney N. L. H.; Whale T. F.; Guo Q.; Gibson M. I. Cryopreservation and Rapid Recovery of Differentiated Intestinal Epithelial Barrier Cells at Complex Transwell Interfaces Is Enabled by Chemically Induced Ice Nucleation. ACS Appl. Mater. Interfaces 2024, 16 (18), 23027–23037. 10.1021/acsami.4c03931. 38671549 PMC11082836

[ref27] Pummer B. G.; Bauer H.; Bernardi J.; Bleicher S.; Grothe H. Suspendable Macromolecules Are Responsible for Ice Nucleation Activity of Birch and Conifer Pollen. Atmos. Chem. Phys. 2012, 12 (5), 2541–2550. 10.5194/acp-12-2541-2012.

[ref28] Pummer B. G.; Budke C.; Augustin-Bauditz S.; Niedermeier D.; Felgitsch L.; Kampf C. J.; Huber R. G.; Liedl K. R.; Loerting T.; Moschen T.; Schauperl M.; Tollinger M.; Morris C. E.; Wex H.; Grothe H.; Pöschl U.; Koop T.; Fröhlich-Nowoisky J. Ice Nucleation by Water-Soluble Macromolecules. Atmos. Chem. Phys. 2015, 15 (8), 4077–4091. 10.5194/acp-15-4077-2015.

[ref29] Murray K. A.; Gibson M. I. Post-Thaw Culture and Measurement of Total Cell Recovery Is Crucial in the Evaluation of New Macromolecular Cryoprotectants. Biomacromolecules 2020, 21 (7), 2864–2873. 10.1021/acs.biomac.0c00591. 32501710 PMC7362331

[ref30] Daily M. I.; Whale T. F.; Kilbride P.; Lamb S.; John Morris G.; Picton H. M.; Murray B. J. A Highly Active Mineral-Based Ice Nucleating Agent Supports in Situ Cell Cryopreservation in a High Throughput Format. J. R. Soc. Interface 2023, 20 (199), 20220682 10.1098/rsif.2022.0682. 36751925 PMC9905984

[ref31] Tomás R. M. F.; Dallman R.; Congdon T. R.; Gibson M. I. Cryopreservation of Assay-Ready Hepatocyte Monolayers by Chemically-Induced Ice Nucleation: Preservation of Hepatic Function and Hepatotoxicity Screening Capabilities. Biomater. Sci. 2023, 11, 7639 10.1039/D3BM01046E. 37840476 PMC10661096

[ref32] Müllers Y.; Meiser I.; Stracke F.; Riemann I.; Lautenschläger F.; Neubauer J. C.; Zimmermann H. Quantitative Analysis of F-Actin Alterations in Adherent Human Mesenchymal Stem Cells: Influence of Slow-Freezing and Vitrificationbased Cryopreservation. PLoS One 2019, 14 (1), e0211382 10.1371/journal.pone.0211382. 30682146 PMC6347223

[ref33] Acker J. P.; Croteau I. M. Pre- and Post-Thaw Assessment of Intracellular Ice Formation. J. Microsc. 2004, 215 (2), 131–138. 10.1111/j.0022-2720.2004.01375.x. 15315499

