# Supplementary material for: Induced Extracellular Ice Nucleation Protects Cocultured Spheroid Interior and Exterior during Cryopreservation
Source: ACS Biomater Sci Eng. 2024 Sep 24;11(1):208–12. doi: 10.1021/acsbiomaterials.4c00958 (PMC11733914; doi:10.1021/acsbiomaterials.4c00958)
Supplement: Supplementary file 1 — ab4c00958_si_001.pdf [file ab4c00958_si_001.pdf]

Supporting information

Induced extracellular ice nucleation protects co-cultured spheroid interior and exterior during cryopreservation

Yanan Gao, [a,f] Akalabya Bissoyi,[d] Qiongyu Guo,[f] and Matthew I. Gibson[a,b,c,d*]

a) Department of Chemistry, University of Warwick, Coventry, CV4 7AL, United Kingdom

b) Division of Biomedical Sciences, Warwick Medical School, University of Warwick, Coventry, CV4 7AL, United Kingdom

c) Department of Chemistry, University of Manchester, Oxford Road, Manchester, M13 9PL, UK

d) Manchester Institute of Biotechnology, University of Manchester, 131 Princess Street, Manchester, M1 7DN, UK

f) Department of Biomedical Engineering, Southern University of Science and Technology, Shenzhen, Guangdong 518055, China

Corresponding Author Email, matt.gibson@manchester.ac.uk

Experimental

All materials were used as supplied unless otherwise stated.

Cells: Human Caucasian lung carcinoma cells (A549), A549-GFP was purchased from CellBioLab, inc., (UK, AKR-209). Human Hepatocellular carcinoma (HepG2, Loughborough, UK, ECACC85011430).

Materials: Dulbecco's phosphate buffered saline (DPBS, Thermo Fisher Scientific, D8537), Ham's F-12K (Kaighn's) Medium (Gibco, Paisley, UK), non-US origin fetal bovine serum (FBS) (Sigma Aldrich, Dorset, UK, F7524) and PSA (HyClone, Cramlington, UK), and Minimum Essential Medium Eagle medium (Sigma Aldrich, M4655) were purchased for cell culture. 0.25% trypsin plus 1 mM EDTA (Gibco, 25200072), Dulbecco's Modified Eagle Medium (DMEM), Gibco Sodium Pyruvate (12539059), Gibco™ MEM Non-Essential Amino Acids Solution (100X) (11140050) were purchased at Gibco.

U-bottom 96-well clear ultra-low attachment microplates (10023683) were from Corning company. Agarose Bio Reagent for molecular biology (Sigma, A9539), sterile saline [0.9% (w/v) NaCl], WST-1 proliferation Assay (abcam, Cambridge, UK), Carpinus betulus (hornbeam) pollen (CARB.0116, Pharmallerga, Lisov, Czech Republic) were used for spheroids' experiments. Corning CoolCellTM LX cell freezing vial container (CLS432001), Live-Dead Viability/Cytotoxicity kit (2326049), Invitrogen ActinGreenTM 488 ReadyProbeTM reagent (R37110), Hoechst 33342 solution (62249) and cryovials were all purchased from Thermo Fisher (Loughborough, UK). 10% dimethyl sulfoxide hybrid-max, sterile-filered (D2650), Triton X-100 and 0.4 % trypan blue (T8154), doxorubicin hydrochloride 98% (DOX, 860360) were purchased from Sigma Aldrich Co Ltd.

Equipment: Incubator (Haier Biomedical, HCP-168, UK), centrifuge for 15 mL tubes (VF4G003085), Invitrogen ™ Countess™ 3 FL Automated cell counter, BioTek Synergy HT microplate reader, an inverted microscope (Olympus, Southend-on-Sea, UK), VIA freeze (Asymptote Ltd, Cambridge, UK), -80 °C freezer (Thermo Scientific™).

Methods

Cell culture

Human Caucasian lung carcinoma cells (A549) from the European Collection of Authenticated Cell Cultures (ECACC) (Salisbury, UK) were seeded at density of 1.5×10^6 cells per T175 cell culture Nunc flasks (Corning, NY) using 40 mL of Ham's F-12K (Kaighn's) Medium (Gibco,

Paisley, UK) supplement with 10% fetal bovine serum (FBS) (Sigma Aldrich, Dorset, UK) and 1% PSA (HyClone, Cramlington, UK). Cells were kept in an incubator in a humidified atmosphere containing 5% CO_2 at 37 °C. After over 90% confluency, the subconfluent in T175 was washed with DPBS (Thermo Fisher Scientific). 10 mL of 0.25% trypsin plus 1 mM EDTA (Gibco) was used for dissociation for 5 min. 10 mL of total media was subsequently added to the flask to end enzymatic reaction. The solution was then centrifuged at 2000 rpm for 5 min. The cells were grown at 37 °C with 5% CO_2 in air. 2×10^5 cells per well of 24 well-plate were seeded of each type of cell before cryopreservation.

The medium for A549-GFP cells based on Dulbecco's Modified Eagle Medium (DMEM) with 10% (v/v) fetal bovine serum (FBS), 1% (v/v) penicillin-streptomycin, and additional components supplements 1.0 mM Gibco Sodium Pyruvate, 1% (v/v) essential amino acids (NEAA).

Human Hepatocellular carcinoma (HepG2) cells were cultured using Dulbecco's minimal essential medium (DMEM) supplemented with 10% fetal bovine serum, penicillin (100 units/ml), and streptomycin (100 µg/mL) at 37 °C with 5% CO_2.

Fabrication of spheroids

Prepare agar moulds using 3D Petri Dishes®

1 g of pure agarose powder (Sigma) added in 50 mL of sterile saline [0.9% (w/v) NaCl] in a dry 100 mL autoclave-safe glass bottle, autoclave for 30 minutes. Placing the bottle in a microwave oven to boil the solution. Pipetting 500 µL of agarose solution in a 3D Petri Dishes®, leave it for 10 min for gelation. The agarose micro-moulds were subsequently removed from the 3D Petri Dishes, and transferred into a 12-well plate and equilibrating with 1 mL of cell culture medium each well for 1 hour in an incubator.

Formation of A549 or HepG2 spheroids

Remove all culture medium from the wells before seeding cells in each micro-mould. 190 µL of A549 or HepG2 cells suspension was placed in each chamber containing indicated concentrations of cells (1000~8000 cells/spheroid). Plates were placed in an incubator for 30 min and then adding 2.5 mL medium to each well before put back in an incubator, medium was replaced twice during 7 days. A549 cells were grown in F-12 K complete medium with 10% (v/v) FBS, 1% antibiotic-antimycotic solution 100x, while HepG2 cells were cultivated using MEM supplemented with 10% (v/v) FBS, 1% antibiotic-antimycotic solution 100x, 1% MEM

Non-Essential Amino acid solution 100X. An inverted microscope (Olympus, CX41, Southend-on-Sea, UK) were applied to monitor spheroid formation and spheroids images analysis used ImageJ software v1.52.

Development of co-culture spheroids

243,000 of HepG2 or A549 cells per agarose micro-mould were seeded (~3000 cells per spheroid) and settled for 30 min, then162,000 of A549-GFP cells were seeded in each mould (2000 cells per spheroid) in a 12 well plate. Placed the plate in an incubator for 30 min and then adding 2.5 mL medium to each well, grow for 6 days in an incubator. The medium was replaced every second day.

Preparation of cryoprotectant agent (CPA) solution

10% DMSO solution was made with 10% (v/v) DMSO solution, 10% (v/v) FBS and 80% (v/v) basal medium.

The CPA with ice nucleation agent (+IN) contained 50% (v/v) pollen washing water (PWW), 10% (v/v) DMSO solution, 10% (v/v) FBS and 30% (v/v) basal medium. For PWW, 0.8 g of pollen power (Hornbeam, Carpinus betulus) was dissolved in 10 mL of distilled water in a 15 mL centrifuge tube, vortexing the tube for 2 min and placed the tube at 4 °C overnight (24 hours). The solution was filtered using a 0.22 μm filter to remove the pollen or debris. 10 mL of cryoprotectant formulation including 5 mL of sterile pollen solution, 1mL of FBS (10 %) and 1mL of DMSO (10 %), and 3 mL of DMEM based medium.

Nucleation temperature determination within 96 well plate

A 96-well plate was used. Each well was filled with 100 μL of either 10% DMSO solution or 10% DMSO + IN (ice nucleation agent) solution. T-type thermocouples were positioned in the wells, and the plate was then placed in a -80 °C freezer to measure freezing temperature during 1°C/min cooling. The temperature changes were monitored using a PT100 thermocouple logger integrated into the PicoTech PT-104 system. The ice nucleation temperature was determined by identifying the point at which a temperature spike occurred, corresponding to the exothermic nature of ice formation as the temperature drops.

Cryopreservation of spheroids

Spheroids were transferred in cryovials, with 1 mL of CPA containing 10% DMSO inducing ice nucleation agent in each cryovial (Corning, NY) or 10% DMSO alone. 10% DMSO alone group as the control experiment. The vials were placed in CoolCell™ LX cell freezing vial container (15552781, Corning) and were stored in a -80 °C freezer for 24 h to control cooling rate to be frozen gradually.

The vials were rapidly thawed in a 37 °C water bath after taking out from the freezer, until no ice crystal remained. The CPA solution was removed and 1 mL of pre-warmed complete cell culture medium was added in each vial. The spheroids were then transferred into 6-well plates coating with 1 mL agarose, adding 2.5 mL medium for 24 h culture. 6 spheroids were transferred to each well of a low attached 96-well plate to determine the viability after thawing for 24 h. Images of before/after freezing were captured by an Olympus CX41 microscope.

Spheroids viability

Unfrozen and post-thaw spheroids were measured using a WST-1 proliferation reagent (Abcam, ab201119, UK) to determine the viability of 3D spheroids. We picked 6 spheroids with 100 μL media to each well in a 96-well white plate, added 10 μL of the reagent per well. The plate was placed in an incubator for 4 hours. Subsequently, the plates were shake for 1 min to lyse cells, and optical density (OD) was measured by a BioTek Synergy HT microplate reader at 450 and 620 nm wavelength.

The LIVE/DEAD® Viability/Cytotoxicity Assay Kit was used to observe apoptotic cell death and morphology of spheroids. It provides a two-colour fluorescence cell viability assay that is based on the simultaneous determination of live and dead cells with two probes that measure recognized parameters of cell viability- intracellular esterase activity and plasma membrane integrity. For this assay, remove the media from each well, the spheroids were kept in the mould and washed with DPBS for two times. An approximately 2 μM of calcein AM (5 μL) and 4 μM of ethidium homodimer-1 (20 μL) were added to 10 mL of sterile D-PBS in a 15 mL tube with 33 μM Hoechst 33342 (Life Technologies, Carlsbad, CA), vertexing to ensure thorough mixing. Then, 200 μL solution was added in each mould and incubated at room temperature for 1 h, spheroids were then transferred to confocal dishes and captured images using a FV3000 confocal laser-scanning microscope (Olympus, Tokyo, Japan). The polyanionic dye calcein AM retained within live cells, showing an intense uniform green florescence in live cells at ex/em ~495 nm/ ~515 nm. EthD-1 entered cells with damaged

membranes, binding to nucleic acids and producing a bright red fluorescence in dead cells at ex/em ~495 nm/ ~635 nm.

Histology protocol:

Cryosection

The samples were fixed in 4% paraformaldehyde (PFA) at 4 ˚C overnight and replaced the solution with 30% sucrose for 24 hours. The samples were embedded in O.C.T. Compound Mounting Medium for Cryotomy (VWR® Q Path®, 00411243) in the cryomold (Agar Scientific Ltd, UK, AGG4581), and then placed them on dry ice for 30 minutes. Samples were stored in -80 ˚ C before cryosection.

We used cryostat (Epredia™ CryoStar™ NX50) to prepare the cryosection of samples. The sample was attached on the tissue holder of the cryostat with a drop of OCT at room temperature, all samples kept in dry ice before use. Subsequently, the mounted tissue holder was placed at −35°C for 20 minutes (precooling) in the cryostat. 10 μm of sections were cut in the cryostat with a motor-driven microtome using a blade (Epredia™ Ultra Disposable Microtome Blades, MX35, 3053835). Sections were unfolded with a brush and transferred onto PolysineTM microscope slides (VWR International bvba, 631-0107). This was done immediately after the slides were introduced into the cryostat, and placed at room temperature for 10 min before storing them in the storage box.

Hematoxylin and eosin (H&E) staining

The slides with cryo sections followed the protocol of the H&E staining Kit (Generon Ltd, HAE-2). The slides were placed in 70% ethanol for 30 seconds and wash with deionized water. Immerse in fresh Haematoxylin for 1 min and rinse the slides gently in water twice to remove excess hematoxylin. Differentiate the sections in blueing solution for 30 seconds and wash with water twice. The slides were then dried and placed in eosin solution for 1 min. Rinse the slides gently with water to remove excess eosin. Dehydrate sections with 100% ethanol for 30 seconds, then placed in xylene for 30 seconds to remove any remaining water. Apply mounting medium (Sigma-Aldrich, 06522) to the stained sections on the slides and place a coverslip over the sections. Examine the prepared slides and capture images using an Olympus CX41 microscope.

Cytoskeleton Staining

The cryo sections were covered with 0.1% Triton X-100 in PBS for 15 minutes, washing twice with DPBS and then sections were incubated with primary antibody for cytoskeleton dye (Incitrogen ActinGreen ^{TM},488 ReadyProbe reagent overnight at 4 degrees in the fridge. The samples were washed twice with DPBS and then covered with Hoechst 33342 solution for 10 minutes. The slides were covered with coverslips with mounting medium. Images were captured using a FV3000 confocal laser-scanning microscope (Olympus, Tokyo, Japan), ex/em ~ 499/520 nm.

Proteomics analysis

Sample Preparation

Cell Lysis: Spheroids were washed with ice-cold phosphate-buffered saline (PBS) to remove excess media and breaking down the cells to release proteins by RIPA lysis buffer, which was prepared with protease inhibitor. The volume of RIPA buffer is 100 µL per 10^6 cells. A pipette was used to disrupt the cells thoroughly. The samples need to keep on ice to minimize protein degradation. Incubate the lysate on ice for 30 minutes, intermittently vertexing gently. **Protein Extraction**: Samples were sonicated in high setting in ice bath for 2 min. Centrifuge the lysate at a high speed (12,000 x g) for 20 minutes at 4°C to pellet cell debris and nuclei. Transfer the supernatant (containing solubilized proteins) to a new, chilled microcentrifuge tube. **Protein quantification**: the concentration of extracted proteins was measured using a Nanodrop 2000/2000c (Thermo Fisher Scientific, USA).

Protein Digestion

Buffer exchange: Samples were added into the filter units with 400 µL of 50mM ammonium bicarbonate (ABC), centrifuge 8000xg for 20 min, and repeat this step three times. **Reduction and alkylation in ABC**: 400 µL of 10 mM TCEP and 40 mM CAA in ABC were added in the tubes for 30 min at room temperature. The solution was then removed by centrifuge 8000xg for 20 min. 400 µL of 50 mM ABC was added in each tube to wash filter and centrifuge 8000xg for 20 min. **Protein digestion**: Trypsin was used to cleave proteins into smaller peptides. 2 µg of trypsin per 100 µg of protein in 400 µL of 50 mM ABC overnight at 37 ˚ C. **Peptide elution**: Transfer filters to new collection tubes, centrifuge 8000xg for 20 min. Then, 400 µL of water was added in each tube and centrifuge for 20 min. All the solution were collected into 2 mL tubes and placed in the centrifuge 1000xg at 60 ˚ C for 1.5 h to evaporate water. 50 µL of finial

solution added in the tubes and samples are ready for mass spectrometry analysis. Data processing and analysis by Scaffold 5.3.0.

Statistical Analysis: Origin 2022b, ImageJ v1.52 and GraphPad Prism 9 software were used to analyse the data. To determine significance between the means of two groups, an unpaired two-sided T-test was conducted using GraphPad Prism 9 Software.

Additional Data

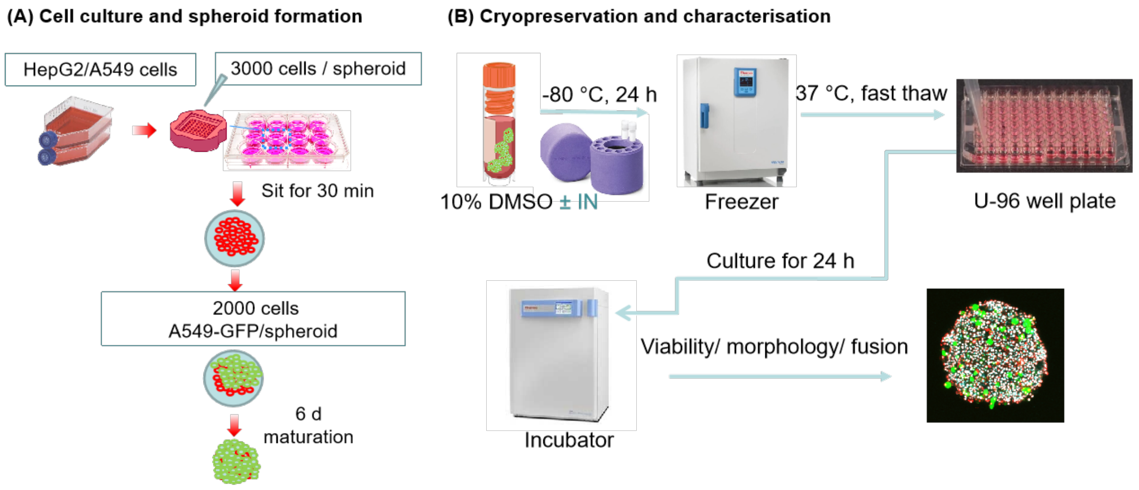

Scheme S1. The process of fabrication of spheroids, cryopreservation and characterisation. (A) Cell culture and spheroids formation with cells for 6 days, red indicated 3000 cells of A549/HepG2 in the core, green indicated 2000 cells of A549-GFP. (B) Cryopreservation and characterisation of spheroids, green indicated A549-GFP cells, red indicated dead cells (Ethidium homodimer-1, EI); white indicated (over exposed) Hoechst 33342.

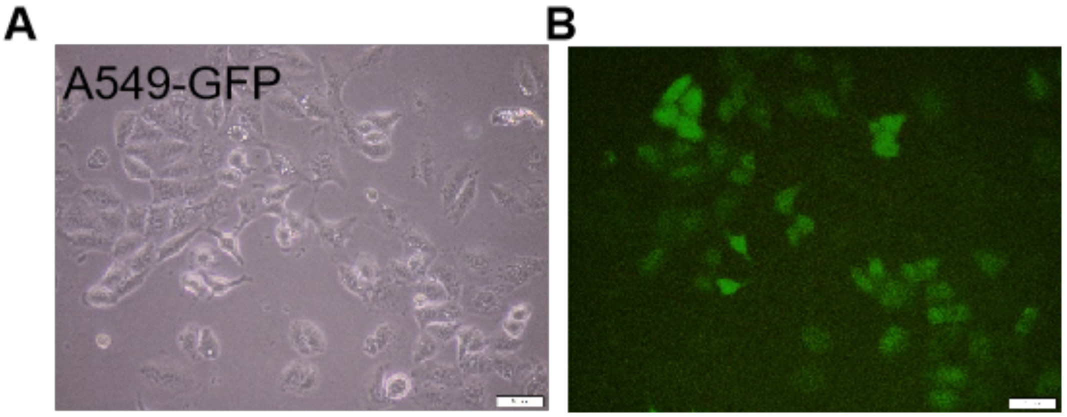

Figure S1. Image of A549-GFP cells. A. Bright field. B. Fluorescence field. Scale bar: 20μm.

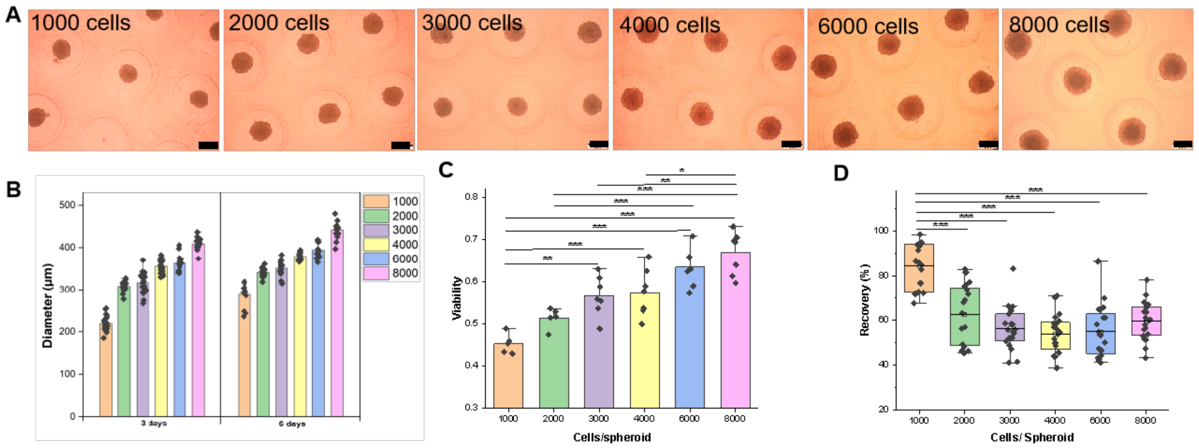

Figure S2. Preparation of HepG2 spheroids with different cell numbers. A. Spheroids with 1000 to 8000 cells. B. Diameters of spheroids at day 3 and day 6. C. Viability of spheroids with different cell numbers at day 6. D. The recovery of spheroids after freeze-thawing process. Scale bar: 200μm.

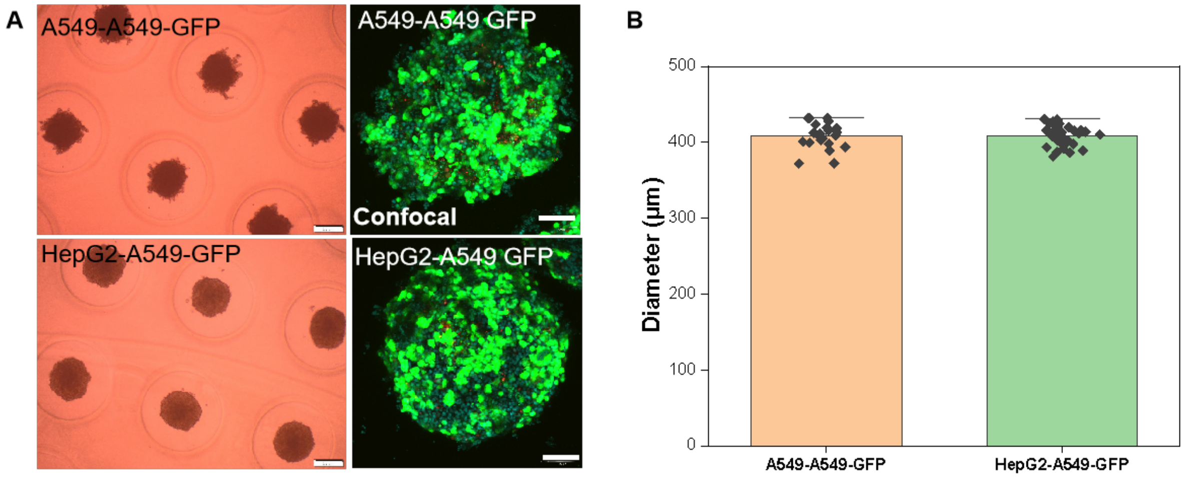

Figure S3. Images of co-culture spheroids. A) Spheroids grown in the agarose moulds for 6 days and the confocal images (Green: A549-GFP cells, Red, Dead cells); B) The diameter of spheroids (A549 3000cells-A549-GFP 2000 cells, HepG2 3000cells-A549-GFP 2000 cells) after growth for 6 days.

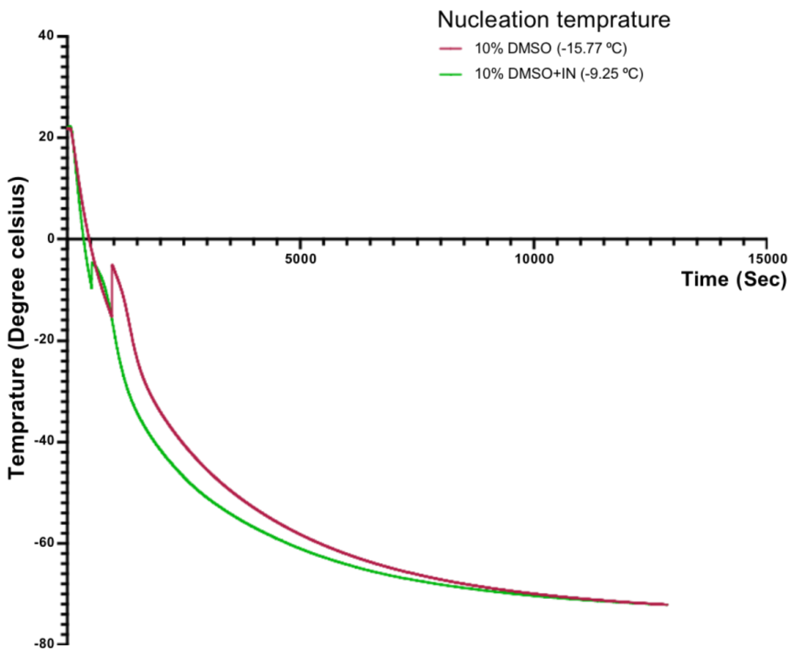

Figure S4. Determination of nucleation temperature. The nucleation temperature of 100 μL of 10% DMSO solution in 96 well plate was –15.77 °C, in contrast, 10% DMSO supplement with IN was –9.25 °C.

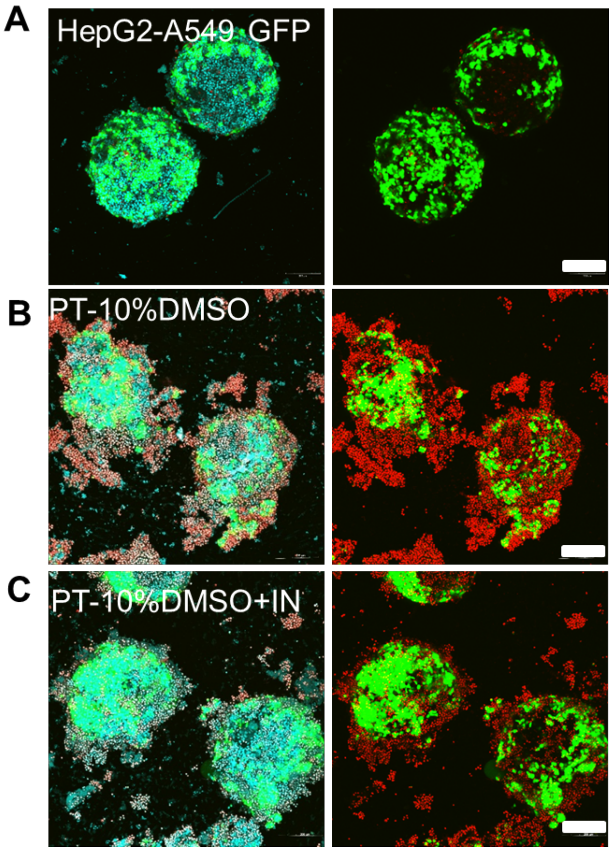

Figure S5. Morphology of spheroids post-thaw 24 hours in 6-well plates. A. Unfrozen spheroids of HepG2-A549-GFP group. B. Post-thaw (PT) spheroids cryopreserved in 10% DMSO. C: Post-thaw (PT) spheroids cryopreserved in 10% DMSO + ice nucleation agent (IN). Green: live A549-GFP cells, red: dead cells, blue: DAPI for nuclei. Left images: merge of three signals, right side images: merge of red and green signals. Scale bar: 200 μm.

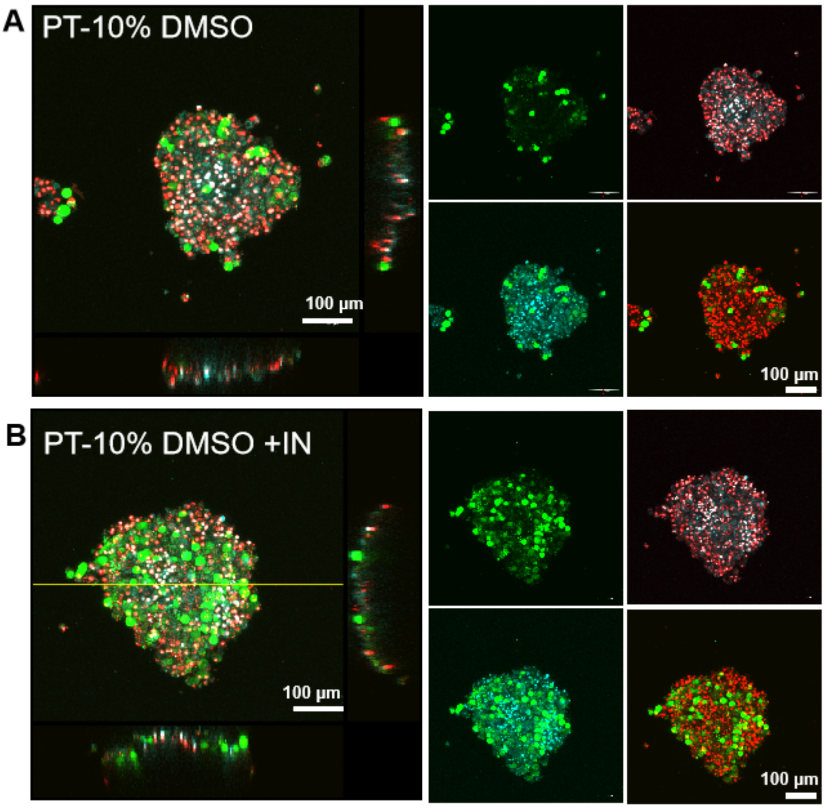

Figure S6. 3D view of confocal images of post-thaw A549-A549-GFP spheroids. Green: live A549-GFP cells, red: dead cells, blue: DAPI for nuclei. Left images: merge of three signals, and the scan depth of the spheroids. Scale bar: 100 μm.

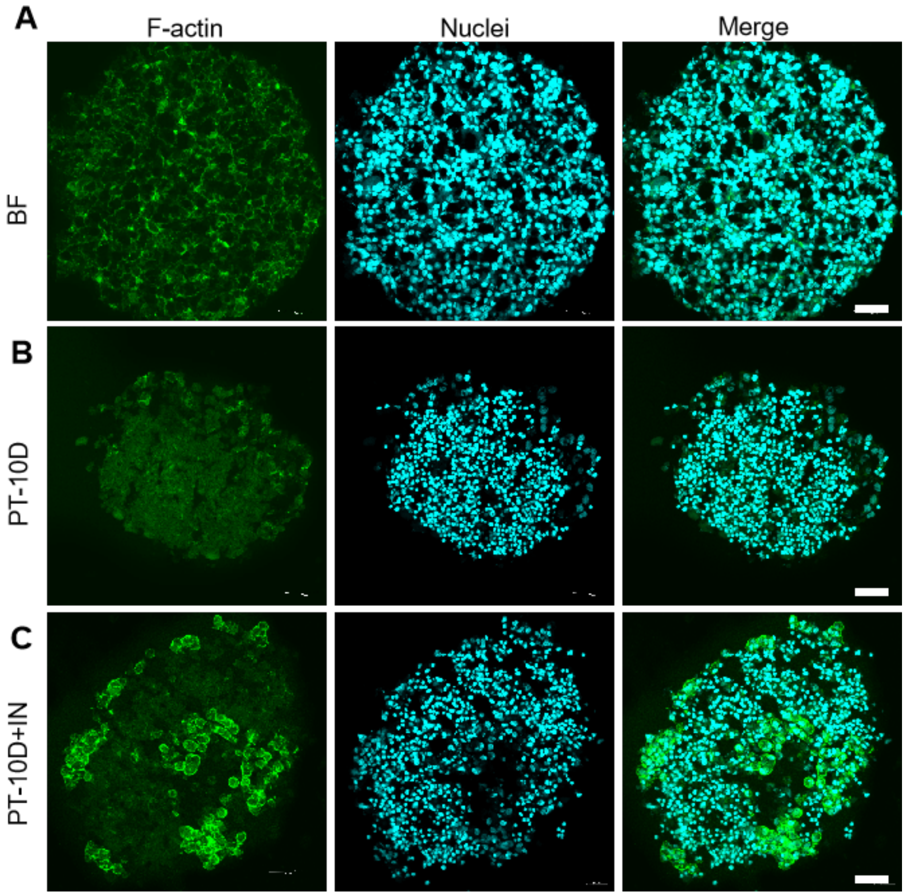

Figure S7. The images of 10 μm of sections of HepG2-A549-GFP spheroids. A. Before freezing (BF). B. Post-thawing spheroids with 10% DMSO as cryoprotectant (PT-10D). C. Post-thawing spheroids with 10% DMSO with ice nucleation agent as cryoprotectant (PT-10D+IN). Green: F-actin dye, blue: DAPI for nuclei. Scale bar: 50 μm.

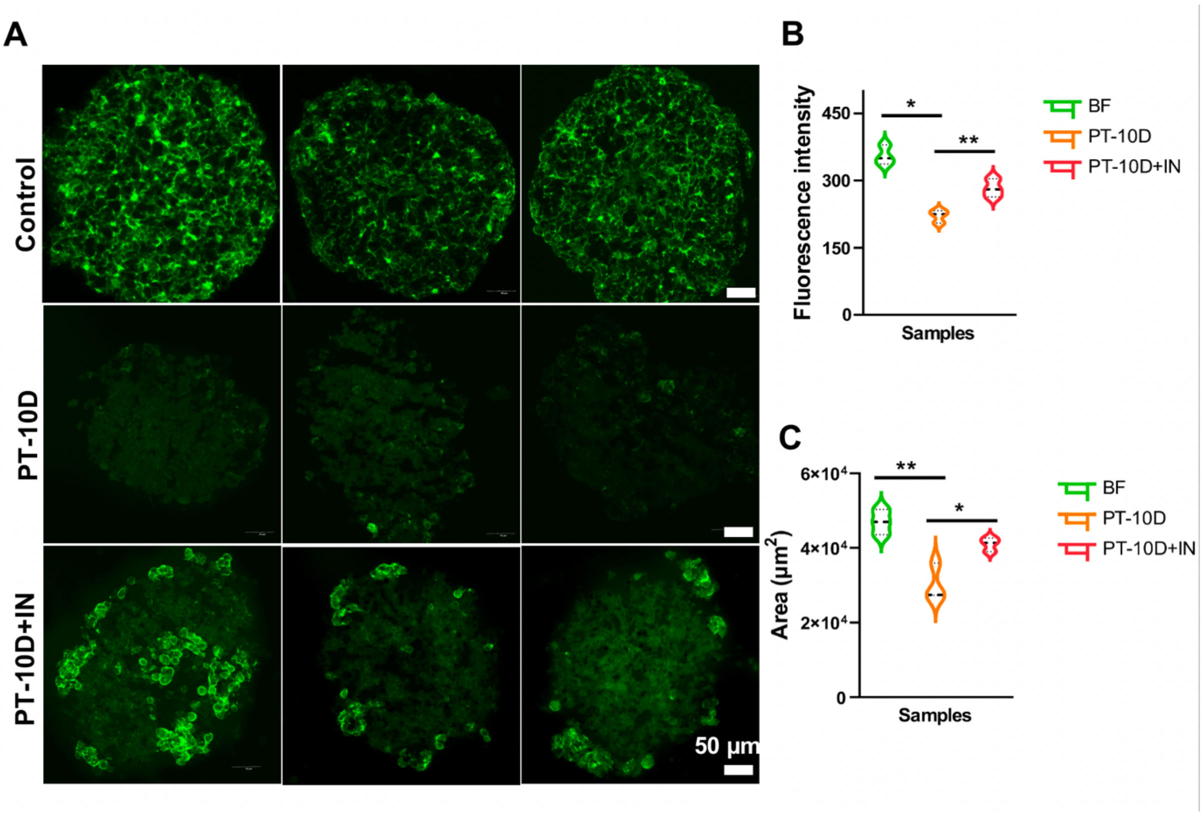

Figure S8. Quantification of actin staining before and after cryopreservation. A. Three images of each condition of spheroids. B-C. Fluorescence intensity and areas of the images were analysed using Image J software.

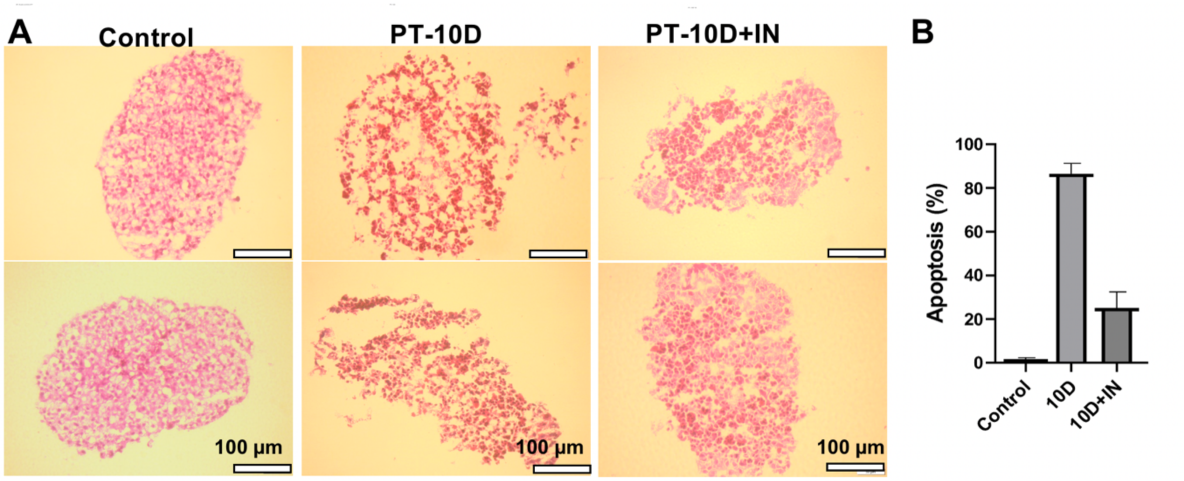

Figure S9. H&E staining images of HepG2-A549-GFP spheroids. BF: Before-freeze. PT-10D: Post-thaw spheroids with 10% DMSO as cryoprotectant. PT-10D+IN: Post-thaw spheroids with 10% DMSO with ice nucleation agent as cryoprotectant. Scale bar: 100 μm.

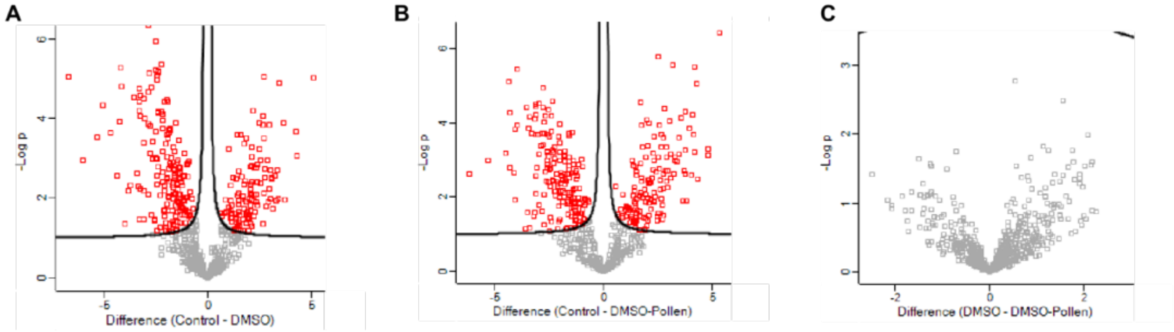

Figure S10. Volcano plots of protein expression. Protein expression difference between the control group and cryopreserved groups of spheroids of HepG2-A549-GFP in DMSO or DMSO+IN. A. Difference of control group and DMSO alone group. B. Difference of control group and DMSO+IN group. C. Difference of DMSO alone group and DMSO+ IN group.

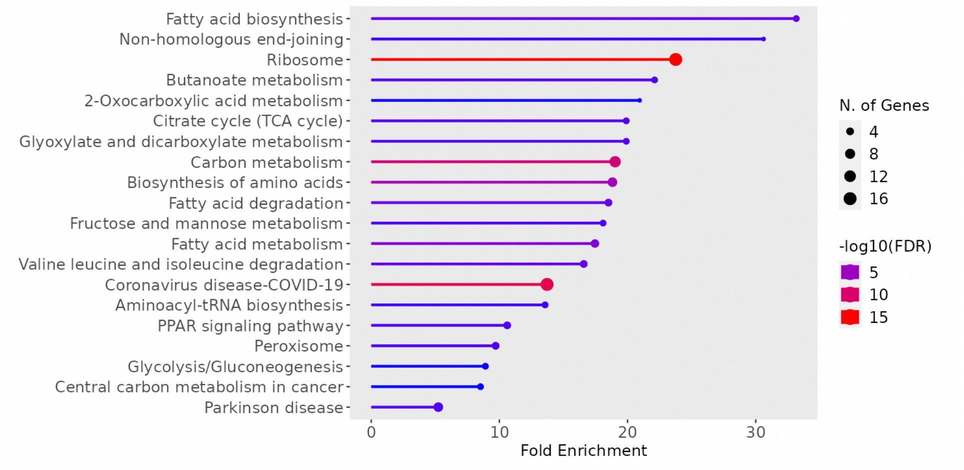

Figure S11. Significant difference of proteins between groups. There were 326 proteins showing significant difference Fatty acid biosynthesis, non-homologous end-joining, ribosome, butanoate metabolism, 2-oxocarboxylic acid metabolism are top five related pathways.

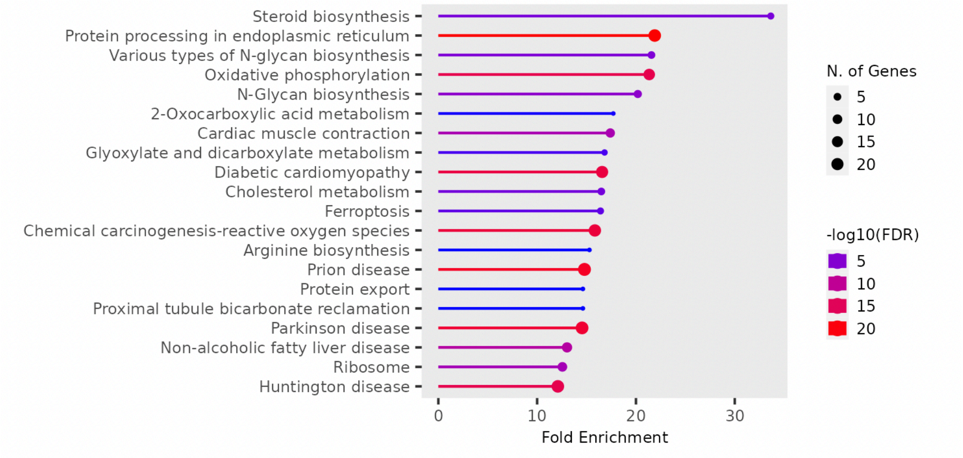

Figure S12. Non-significant difference of proteins between groups. 306 proteins showed no difference, steroid biosynthesis, protein processing in endoplasm reticulum, various types of N-glycan biosynthesis, oxidative phosphorylation, N-Glycan biosynthesis are top five related pathways.
